# Novel 3′-Processing Integrase Activity Assay by Real-Time PCR for Screening and Identification of HIV-1 Integrase Inhibitors

**DOI:** 10.1155/2015/853891

**Published:** 2015-05-07

**Authors:** Supachai Sakkhachornphop, Weeraya Thongkum, Chatchai Tayapiwatana

**Affiliations:** ^1^Research Institute for Health Sciences, Chiang Mai University, Chiang Mai 50200, Thailand; ^2^Division of Clinical Immunology, Department of Medical Technology, Faculty of Associated Medical Sciences, Chiang Mai University, Chiang Mai 50200, Thailand; ^3^Biomedical Technology Research Unit, National Center for Genetic Engineering and Biotechnology, National Science and Technology Development Agency, Faculty of Associated Medical Sciences, Chiang Mai University, Chiang Mai 50200, Thailand

## Abstract

The 3′-end processing (3′P) of each viral long terminal repeat (LTR) during human immunodeficiency virus type-1 (HIV-1) integration is a vital step in the HIV life cycle. Blocking the 3′P using 3′P inhibitor has recently become an attractive strategy for HIV-1 therapeutic intervention. Recently, we have developed a novel real-time PCR based assay for the detection of 3′P activity* in vitro*. The methodology usually involves biotinylated HIV-1 LTR, HIV-1 integrase (IN), and specific primers and probe. In this novel assay, we designed the HIV-1 LTR substrate based on a sequence with a homology to HIV-1 LTR labeled at its 3′ end with biotin on the sense strand. Two nucleotides at the 3′ end were subsequently removed by IN activity. Only two nucleotides labeled biotin were captured on an avidin-coated tube; therefore, inhibiting the binding of primers and probe results in late signals in the real-time PCR. This novel assay has successfully detected both the 3′P activity of HIV-1 IN and the anti-IN activity by Raltegravir and sodium azide agent. This real-time PCR assay has been shown to be effective and inexpensive for a high-throughput screening of novel IN inhibitors.

## 1. Introduction

Integration of transcribed double-stranded DNA into a host chromosome is a vital step for retrovirus replication [1, 2]. This requires the integrase (IN) enzyme to cleave highly conserved sequences at each viral long terminal repeat (LTR). IN removes two adjacent nucleotides, guanine (G) and tyrosine (T), from the 3′ end of U5 or U3, the so-called 3′-end processing (3′P). Subsequent to viral RNA transport into the nucleus, IN then catalyzes the joining of the processed DNA into the host chromosome [1–4]. Therefore, 3′P reaction is proficient process (80% of linear DNA is processed) happening in cytoplasm at the early stage of the replication cycle, related to or rapidly after reverse transcription [5]. Stalling the 3′P using 3′P inhibitor to eliminate the fate of virus has newly converted a gorgeous approach for HIV-1 therapeutic intervention.

HIV-1 IN has been a validated target for treatment of persons infected with HIV. Over the past two decades, several IN inhibitors that interrupt HIV-1 DNA integration have been identified for potential use in HIV treatment. Among HIV-1 IN inhibitors, Raltegravir (RAL; Isentress, Merck) was the first integrase strand transfer inhibitor (INSTI) licensed in 2007 by the U.S. Food and Drug Administration (FDA) for treatment in HIV-1 infected individual [6]. Another INSTI, dolutegravir (Tivicay, ViiV Healthcare, GlaxoSmithKline), was found to be an antiviral inhibitor in advanced clinical trials [7] and was licensed in 2013. Resistance to such drugs has been reported as a result of IN mutations [8, 9]. Up to date, 3′ P inhibitors have not been introduced to the market. In contrast to antiretroviral (ARV) drugs, the alternative strategy to inhibit HIV-1 integration such as zinc finger protein has been reported for its potential application in gene therapy [10].

Standard methods to detect and measure HIV-IN activity require the use of isotope-labeled DNA as the substrate and denaturing gel separation [11, 12], but such techniques are time-consuming and troublesome for screening several inhibitor candidates. As a result, nonradioisotopic assays, including many fluorescence-based, have been developed and used for high-throughput screening of HIV-IN inhibitors [13, 14]. These techniques still require multiple labeling of oligonucleotide substrates, as well as advanced technology not readily available in most laboratories.

We report the design and testing of a nonradioisotopic, real-time polymerase chain reaction (PCR) assay to determine HIV-1 IN activity, which could be applied for screening of novel antiviral agents. The biotinylated-LTR of HIV-1 DNA was designed accordingly as a substrate for cleavage by IN. Blocking of IN for cleaving of DNA substrate and the remaining of the unprocessed LTR substrate can be detected using a specific primer and probe in real-time PCR. The efficacy of the anti-IN agent to inhibit IN activity contributes to a low signaling in real-time PCR. The assay has successfully been shown to measure the IN enzymatic activity. It is proven simple and could be adapted for a high-throughput screening of novel 3′-processing IN inhibitors. Disruption of* in vitro* IN enzymatic activity could be an attractive method applied for screening of novel antiviral agents.

## 2. Results

### 2.1. Principle of Real-Time PCR for IN Activity Assay

**Figure 1:**
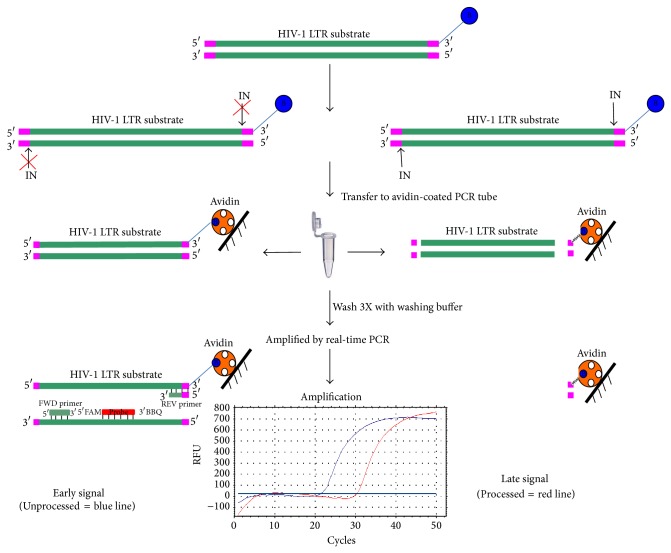
Schematic diagram showing the principle of the 3′-processing integrase activity assay based on real-time PCR. HIV-1 LTR substrate is labelled with biotin at 3′ end of sense strand. IN has activity to cleave dinucleotide from the LTR oligonucleotide at 3′ end at CA recognition motif. After washing step, only the processed biotinylated GT-dinucleotide (pink colour) binds to avidin-coated tube. The unprocessed biotinylated HIV-1 LTR substrates regarding IN activity can be amplified and detected by specific primers and probe. Early signal for unprocessed reaction is represented by blue line, whereas late signal for processed reaction is represented by red line.

The reaction of real-time PCR for IN activity assay is portrayed schematically in Figure 1. To determine 3′ processing of HIV-1 IN, a biotinylated double strand of 100-mer oligonucleotides and IN enzymes were added to bind to the avidin-coated tube. IN can specifically cleave a biotinylated dinucleotide from the LTR oligonucleotide at the CA recognition motif at the 3′ end. Following the washes, the processed biotinylated GT-dinucleotide remained bound to avidin-coated tube. For screening of inhibitors, an array of candidate IN inhibitors was added to the reaction and the remaining of unprocessed biotinylated HIV-1 LTR substrates was then amplified and detected by a specific primer and probe.

### 2.2. Integrase Expression and Purification

**Figure 2:**
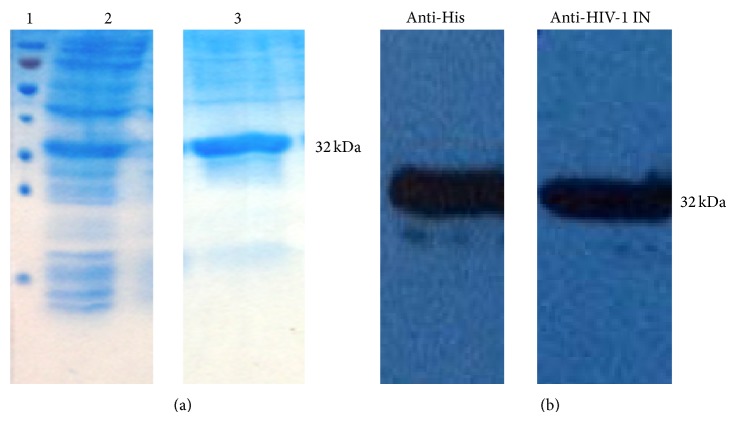
Purification of integrase enzyme. (a) Purified protein fractions were analyzed by SDS-PAGE and stained with Coomassie Blue. Lane 1: molecular weight markers; 2: crude extracts; 3: fraction of HIV-1 IN eluted from the His Trap column revealed minor contaminating species. (b) Purified IN was performed by the Western blotting by using anti-His mAb and anti-HIV-1 IN mAb as primary antibody.

Integrase was purified from the soluble fraction (Figure 2(a)). Lane 2 [total lysate of* E. coli* with pINSD.His.sol was induced by IPTG after the O.D._600_ reached 0.8 at 30°C for 3 hrs.] indicates a successful expression of IN enzyme (approximately 32 kDa). Lane 3 shows that the fusion protein was bound to the chromatography column and IN was eluted by elution buffer.

Western blotting was performed using anti-His mAb and anti-HIV-1 IN mAb as primary antibody (Figure 2(b)); the analysis demonstrated that IN-His_6_ (approximately 32 kDa) was successfully expressed.

### 2.3. Optimization of Avidin-Coated PCR Tube

**Figure 3:**
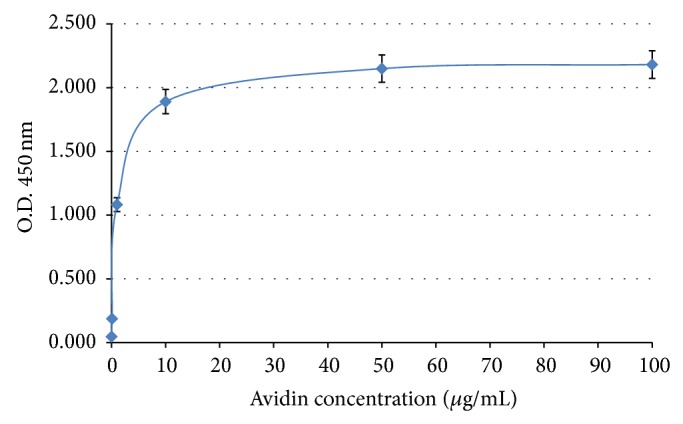
The optimization of avidin concentration for coating the PCR tubes. Avidin was diluted in PBS buffer to a final concentration ranging from 0.1, 1, 10, 50, and 100 *μ*g/mL, respectively. Biotin-HRP was added to monitor the amount of avidin in the PCR tubes. H_2_O_2_ substrate was used and 1 N HCl was used to stop and read the optimum density (O.D.) at 450 nm.

Since varying concentrations of avidin were used for coating the PCR tubes, optimized concentration of avidin needed was determined using biotin-HRP as shown in Figure 3. The binding of biotin-HRP to avidin-coated plate reached a plateau when the concentration of avidin was greater than 50 *μ*g/mL. Therefore, 100 *μ*g/mL of avidin was selected as an optimal concentration for coating of PCR tube for the consequent experiments.

### 2.4. Integrase Activity in Conventional PCR

**Figure 4:**
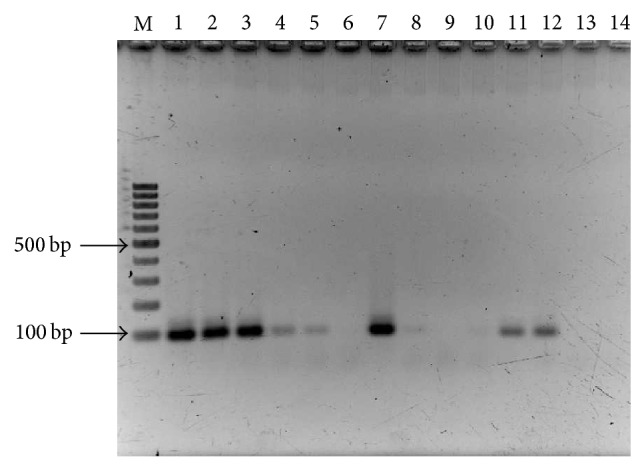
Integrase activity in conventional PCR. Biotinylated HIV-1 LTR substrates were added in reaction mixture (lanes 1–12) to a final concentration at 10 pM (lanes 1, 4, 7, and 10), 1 pM (lanes 2, 5, 8, and 11), and 0.2 pM (lanes 3, 6, 9, and 12), respectively. Lanes 1–3: the reaction mixture without IN; lanes 4–6: the reaction mixture with IN; lanes 7–9: the reaction mixture with IN and Raltegravir to a final concentration at 10 *μ*M; lanes 10–12: the reaction mixture with IN and NaN_3_ to a final concentration at 5%; lanes 13-14: IN and reaction buffer only as negative control.

Integrase activity was determined using biotinylated HIV-1 LTR substrates that mimic the sequences of U5 end of HIV-1 DNA. Raltegravir was used as a positive control for inhibition of IN activity. IN enzyme in the reaction cleaved the 3′-end LTR and two nucleotides (GT) labeled biotin were removed from 3′ end. The reaction was transferred into an avidin-coated tube. These resulting two nucleotides could be captured in tube and inhibited binding by primer, thus resulting in an absence of amplification product. Our result showed that the optimum concentration of biotinylated HIV-1 LTR substrates was 0.2 pM to completely differentiate between the unprocessed (lane 3) and processed activities by IN enzyme (lane 6) in Figure 4. PCR products were observed when incubating 10 *μ*M Raltegravir with IN and with 10 and 1 pM of biotinylated LTR substrates (lanes 7 and 8, resp.). PCR products were seen in the reaction using NaN_3_ as integrase inhibitor in lanes 10–12.

### 2.5. Development of 3′-Processing IN Activity Assay by Real-Time PCR

**Figure 5:**
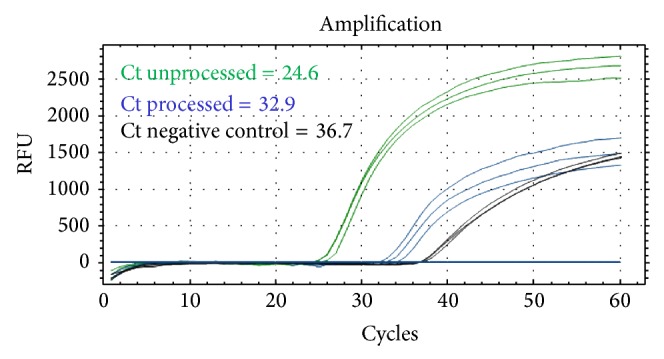
The 3′-processing IN activity assay by real-time PCR. Biotinylated HIV-1 LTR substrates were incubated with or without IN enzyme in a reaction tube at 37°C for 16 h. The reaction was transferred to an avidin-coated real-time PCR tube. After washing 3 times, the remaining of unprocessed biotinylated HIV-1 LTR substrates was then amplified and detected by specific primers and probe as described above. The reaction mixture without IN is shown in green line. The reaction mixture with IN is shown in blue line. Negative control is shown in black line. Ct: cycle threshold.

**Figure 6:**
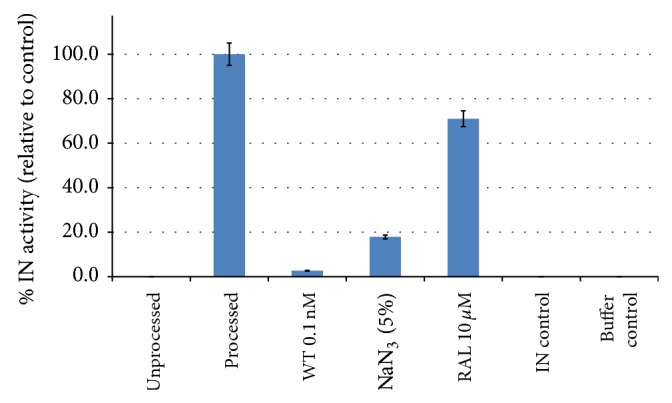
Percentage of IN activity relative to control. The reactions were performed from three independent experiments, each performed in triplicate. The measurements of IN activity were normalized by setting the competitor/inhibitors-free control (processed) and the IN-free control (unprocessed) as 100% and 0%, respectively. Percentage of IN activity was calculated by comparing the percent of IN activity relative to the competitor/inhibitors-free control.

To detect and monitor the 3′ processing of IN activity in real-time platform, biotinylated HIV-1 LTR substrates were incubated with or without IN enzyme in a reaction tube and then transferred into an avidin-coated real-time PCR tube. The remaining of unprocessed biotinylated HIV-1 LTR substrates was then amplified and detected by specific primers and probe as described above. The cycle threshold (Ct) was shown in the early signal (mean Ct = 24.6) in the reaction without IN enzyme, whereas the late cycle threshold (mean Ct = 32.9, and 36.7) can be observed in the reaction with IN enzyme and negative control, respectively (Figure 5). Coincubation with unlabeled competitive LTR-wild type (WT) at 0.1 nM almost completely blocked the signal. In addition, dilution effect was seen in diluted competitor (data not shown). We also observed some partial inhibition of the IN activity by 10 *μ*M Raltegravir and 5% NaN_3_ [15] (Figure 6).

## 3. Discussion

The development of IN inhibition-based anti-HIV therapeutics has been explored following the first drug (Raltegravia, RAL) approval for treatment in HIV-1 infected individuals [6]. However, three major resistant patterns were reported for RAL: E92QV/N155H, T97A/Y143CHR, and G140CS/Q148HKR [16, 17]. Elvitegravir (EVG) has also been shown very potent efficacy to inhibit HIV-1 IN in clinical trial [18, 19]. On the other hand, while the 3′-end processing activity of HIV-1 IN plays a key role during earlier incorporating of viral DNA into the host DNA [1–4], the 3′P inhibitors have not been delivered to the market. Therefore, development of assays for selection of novel 3′P inhibitor compounds deserves attention for HIV drug discovery endeavor.

Traditionally, a high sensitivity of IN activity assay has been performed by gel-based assay utilizing radioisotope in the system. The advantage of this technique was using a small amount of purified IN. However, the methods were laborious and time-consuming [11]. Recently, nonradioactive HIV-1 IN activity assays such as fluorescence-based assays have been developed [14, 20, 21]. However, these techniques required multiple labeling of oligonucleotide substrates. This advanced technology requires expensive machine, which is not readily available in most limited-resource laboratories.

Herein, we reported a real-time PCR based assay specific for 3′P activity of IN. This method is proven simple, reproducible, high-throughput, and efficient for screening for novel IN inhibitor compounds. The improvement of this assay includes the elimination of radioactive handling and the real-time monitoring of results compared with standard gel-based assays. This assay has been optimized in respect to the amount of IN, LTR substrate, and all buffers used in the reaction. The signals of the background in real-time PCR were represented for IN, LTR substrate, and buffer alone. Our assay was validated using RAL, since neither RAL nor EVG inhibits the 3′-processing IN activity even at micromolar concentration. With its synergistic antiviral activity, the existing strand transfer inhibitions (STIs) can be used as positive control for the 3′-processing IN activity in this study [22]. We also incorporated the use of unlabeled competitive LTR-wild type (WT) 100 bp for observing the dilution effect of IN inhibition. NaN_3_ has previously been shown to inhibit the IN activity [15] which is in concordance with our assay's finding. Taken together, we believe that this proposed assay would be highly desirable for the identification of novel 3′-processing IN inhibitor compounds.

Developments in small-molecule therapeutics for HIV-1 such as 93del, 112del aptamers [23], and T30177 (zintevir) [24] have been dramatically inhibited HIV-1 IN in nanomolar level. In addition, there has been evaluated in compounds extracted from natural products for anti-HIV-1 IN activity, such as the active compound from* Pometia pinnata* and* Mimusops elengi* leaves [25, 26]. These compounds showed satisfactory anti-HIV-1 IN activity by cell-free assay system [23] and the ELISA based method described by Tewtrakul et al. [27]. With the achievement of our assay, it can be expected that the assay would be used for compounds screening and development of alternative therapeutics in the near future.

Moreover, for drug discovery in IN mutants therapy, since primary mutation, for example, R263 K can often adversely influence IN enzymatic activity and subordinate viral replication capacity, while secondary mutations compensate for this by growing level of drug resistance consecutively restoring viral fitness [28]. Therefore, for further clinical application, this assay may be useful for drug discovering in IN drug resistance stains by cloning of mutant stains derived from patients and screening for effective therapeutic IN inhibitors.

## 4. Materials and Methods

### 4.1. Oligonucleotide HIV-1 LTR Substrates

Double-stranded DNA analogous of HIV-1 U5 sequence was prepared as a substrate for IN enzyme. A pair of 100-mer oligonucleotides was synthesized: (sense LTR-B) 5′-ACTGGATGGG TGGTTAGACC AGATCTGAGC CTTGTTGTGT GACTCTGGTA CCTAGAGATC CCTCAGACCC TTTTAGTCAG TGTGGAAAAT CTCTAGCAGT-biotin-3′, and antisense LTR was a complementary sequence without biotin labeling. The annealing of HIV-LTR substrates was carried out by mixing an equal volume of sense LTR-B and antisense LTR in DNAse/RNAse-free water with final concentration at 1 *μ*M. The mixture was heated at 95°C for 5 min and slowly cooled to room temperature for 1 hr and kept at −20°C until use.

### 4.2. Integrase Production and Purification

HIV-1 IN was prepared as a recombinant protein. Plasmid vector, pINSD.His.sol, (a gift from NIH AIDS Research & Reference Reagent Program, Germantown, MD) was transformed into* E. coli* strain BL21 (DE3) (Stratagene, La Jolla, CA). Bacteria were cultured in super broth containing 100 *μ*g/mL of ampicillin at 37°C and 200 rpm until optical density of culture was 0.8. Protein expression was induced by the addition of isopropyl-1-thio-*β*-D-galactopyranoside (IPTG) (Fermentas, Burlington, ON, Canada) to final concentration of 0.4 mM and incubated for 3 hrs. Bacterial cell pellets were resuspended in lysis buffer [1 M NaCl, 20 mM HEPES pH 7.5, 2 mM *β*-mercaptoethanol, 0.3 mg/mL lysozyme, 5 mM imidazole] [29]. The bacterial mixture was then incubated at 4°C for 30 min and further lysed by ultrasonication in an ice bath. The lysate was centrifuged at 10,000 rpm for 30 min at 4°C. The supernatant was collected and filtered with microfiltration membranes of 0.22 *μ*M pore size (Merck Millipore Ltd., Co, Ireland). Protein was purified by His Trap column (GE Healthcare Bio-science AB, Uppsala Sweden) using the ÄKTAprime plus system (GE Healthcare Bio-Sciences, Piscataway, NJ). IN was eluted by elution buffer containing 500 mM imidazole, 1 M NaCl, 2 mM *β*-mercaptoethanol, 25 mM HEPES, pH 7.5, and 10% W/V of glycerol. Protein was then dialyzed in 0.5 M NaCl, 20 mM HEPES pH 7.5, 2 mM *β*-mercaptoethanol, 0.3 mM imidazole, 10% (W/V) glycerol for long-term storage at −80°C. The protein concentration was measured by NanoDrop 2000 Spectrophotometer (Thermo Scientific, Rockford, IL) for detection of IN expression. SDS-PAGE, ELISA, and Western blot were used for protein analysis. IN proteins were probed with anti-his monoclonal antibody (Applied Biological materials Inc., Richmond, BC, Canada) and anti-HIV-1 IN (Diatheva, Fano, Italy) as a primary antibody and horseradish peroxidase-labeled goat anti-mouse (KPL, Gaithersburg, MD) as a secondary antibody, and the bands were visualized using SuperSignal West Pico Chemiluminescent substrate (Thermo scientific, Rockford, IL).

### 4.3. Avidin Coating on PCR Reaction Tube

Individual PCR tubes in 8-tube strips (Bio-Rad laboratories, Hercules, CA) were pretreated with 1 mg/mL solution of glutaraldehyde at 56°C for 3 hrs, using 60 *μ*L in each tube [30], and then the tubes were washed with distilled water and 50 *μ*L of (0.1, 1, 10, 50, and 100 *μ*g/mL) avidin (Sigma-ALDRICH, St. Louis, MO) in phosphate buffered saline (PBS) was added and the tubes were incubated at 4°C for 3 days. Unbound avidin was washed out with PBS 2 times and the tubes were flooded with 200 *μ*L of blocking solution (0.05 mol/L Tris-HCl buffer, pH 7.8 containing 0.5% BSA, 0.9% NaCl, and 0.05% NaN_3_) for 1 hr. Then, the tubes were washed with PBS 3 times, dried at 30°C for 3 hrs, capped, and stored at 4°C with silica gel desiccants until use.

To test for efficiency of avidin-coated tubes, the tubes were blocked with 2% BSA in PBS at RT for 1 hr and then washed three times with washing buffer. Then, 0.1 *μ*g/mL biotin-HRP was added to tube. After 1-hour incubation at room temperature, the tubes were washed 3 times with PBS buffer with 0.05% Tween-20. Tetramethylbenzidine (TMB) was used as substrate, and the reaction was stopped using 1 N HCl. The optical density was measured at 450 nm using a microplate reader (AccuReader, Metertech-Inc., Taiwan).

### 4.4. Optimization of Integrase Activity by Conventional PCR

First, 10, 1, and 0.2 pM of biotinylated HIV-1 LTR substrates were incubated with 4 *μ*M of purified IN enzyme in a total volume 50 *μ*L in reaction buffer (25 mM HEPES pH 7.5, 10 mM DTT, 7.5 mM MgCl_2_, 0.05% Nonidet P-40, 30 mM NaCl, and 10 mM MgCl_2_) at 37°C for overnight. Here, 10 *μ*M of Raltegravir was used as a positive control for inhibition of IN activity. After incubation, the mixtures were then transferred into the avidin-coated PCR tube and incubated for 3 min at 37°C and washed 3 times with 250 *μ*L of PBS (pH 7.4). To eliminate washing buffer carryover, a quick centrifugation of the tube is applied. The integrase activity was detected by PCR with the primers SS-F4 (5′-TGGTTAGACCAGATCTGAGCCT-3′) and LTR Rev (5′-ACTGCTAGAGATTTTCCACACTGACTA-3′). Each 25 *μ*L PCR mixture contained 10 *μ*L of 5 Prime MasterMix (×2.5) (5 Prime, Hamburg, Germany), 1 *μ*L of each primer (10 *μ*M), and 13 *μ*L of DNase/RNase-free water, respectively. Each PCR was carried out with an initial denaturation 5 min at 95° followed by 35 cycles of denaturation at 95°C for 30 sec, annealing at 56°C for 30 sec, and extension at 72°C for 30 sec. Terminal elongation was at 72°C for 1 min. The PCR products were run on 2% agarose gel electrophoresis and the gels were stained using ethidiumbromide to verify the band of the PCR product.

### 4.5. 3′-Processing Integrase Assay and Real-Time PCR Conditions

To detect the enzymatic activity of IN by real-time PCR, 10 pM of biotinylated HIV-1 LTR substrates was incubated with 4 *μ*M of purified IN enzyme in a reaction buffer at 37°C for 16 h. The final reaction volume was 50 *μ*L. The reaction was transferred to an avidin-coated real-time PCR tube at 37°C for 3 min for DNA binding and then washed with PBS (pH 7.4) 3 times. A PCR master mix contains* the THUNDERBIRD* Probe qPCR Mix (Toyobo, Japan) with specific primers (SS-F4 and LTR Rev) as previously described above and probe: SupINprobe (5′-6FAM-CTggTACCTAgAgATCCCTCAgAC-BBQ-3′) at final concentration of 175 nM. The reactions were performed on a CFX96 Real-Time System using 20 sec hot start at 95°C, which was followed by 50 cycles of denaturation at 95°C for 3 sec and annealing and extension at 63°C for 30 sec consecutively.

### 4.6. Competitive and Inhibition Effect on IN Assay

To apply this assay to screen for a novel candidate anti-IN agent, the varying concentrations between 5 and 10 *μ*M of unlabeled competitive LTR were used to compete for the IN activity. 10 *μ*M of Raltegravir and 5% sodium azide (NaN_3_) [15] were used as positive controls to inhibit IN HIV-1 LTR activity. The competitor or inhibitors were coincubated with IN and biotinylated DNA substrate before detection of the enzymatic activity in real-time PCR. The reactions were performed as described above.
